# Unresolved questions in the application of artificial intelligence virtual cells for cancer research

**DOI:** 10.1186/s40779-025-00608-0

**Published:** 2025-04-30

**Authors:** Carlos M. Ardila, Pradeep K. Yadalam

**Affiliations:** 1 Department of Basic Sciences, Biomedical Stomatology Research Group, Faculty of Dentistry, Universidad de Antioquia, U de A, 050010 Medellín, Colombia; 2 Department of Basic Sciences, Saveetha Dental College, SIMATS, Saveetha University, Chennai, Tamil Nadu 600077 India

**Keywords:** Artificial intelligence (AI), Cancer, Cells, Oncology

The perspective article by Yang et al. [1], “Build the virtual cell with artificial intelligence: a perspective for cancer research”, provides a compelling vision for the transformative potential of artificial intelligence virtual cells (AIVCs) in oncology. The authors outline how AIVCs could revolutionize cancer research by enabling in silico experimentation, overcoming multi-omics bottlenecks, and accelerating drug development. However, the article presents a comprehensive framework, yet several pivotal questions remain unresolved, which warrant further discussion to advance the field. 

One of the most intriguing aspects of AIVCs is their potential to simulate the dynamic and heterogeneous nature of cancer cells. The authors rightly emphasize the importance of capturing cellular heterogeneity, particularly in the context of genomic instability and clonal evolution. However, the article does not completely address how AIVCs will handle the stochastic nature of cellular processes [2]. For instance, how will AIVCs account for the random fluctuations in gene expression or the probabilistic nature of molecular interactions that are inherent to cellular systems? While the authors propose resolution power as a key metric for model evaluation, it remains unclear how this metric will be operationalized to distinguish between biologically significant heterogeneity and technical noise. Future implementations could leverage stochastic differential equations (e.g., Langevin dynamics) combined with single-cell benchmarking datasets to quantify noise thresholds, while Bayesian neural networks might better capture probabilistic interactions. Could the integration of probabilistic models or stochastic simulations enhance the fidelity of AIVCs in capturing these nuances?

Another critical area that warrants more exploration is the integration of multi-omics data within the AIVC framework [3]. The authors highlight the potential of AIVCs to interpolate between discrete time points, enabling continuous simulation of cellular states. However, the article does not delve into the challenges of integrating disparate data types, such as genomics, transcriptomics, proteomics, and metabolomics, each with its own scale, resolution, and noise characteristics. A potential solution lies in developing hybrid architectures combining graph neural networks (for relational modeling) with attention mechanisms (for scale normalization), validated through perturbation-based consistency checks. How will AIVCs ensure that the integration of these multi-omics datasets does not introduce biases or artifacts that could compromise the accuracy of the simulations? Furthermore, given the rapid evolution of omics technologies, how will AIVCs adapt to incorporate new types of data, such as single-cell multi-omics or spatial transcriptomics, which provide even more granular insights into cellular behavior? Modular pipeline designs with adapter layers for new datatypes, coupled with continuous learning protocols, could maintain model extensibility without catastrophic forgetting.

The article also discusses the potential of AIVCs to generate synthetic data to address the challenge of scarce biological samples, particularly in rare tumors or novel therapeutic contexts. While this is a promising approach, the reliability of generative artificial intelligence (AI) models remains a significant concern. The authors acknowledge the need to balance data generation with maintaining overall data balance, but they do not provide a clear roadmap for achieving this balance. Three key safeguards could be implemented: 1) adversarial validation against held-out experimental data, 2) incorporation of physics-based constraints (e.g., mass-action kinetics) in generative models, and 3) federated learning approaches to prevent bias amplification across institutions. How will AIVCs ensure that the generated data accurately reflect the underlying biological reality, especially for rare or poorly characterized molecular interactions? Additionally, what safeguards will be taken to prevent the amplification of existing biases in the training data, which could lead to misleading conclusions?

In the context of personalized medicine, the authors propose that AIVCs could serve as patient-specific digital models, enabling virtual drug trials and personalized treatment strategies. This is an exciting prospect, but it raises questions about the scalability and generalizability of such models [4]. How will AIVCs handle the vast variability in patient-specific factors, such as genetic background, tumor microenvironment, and comorbidities? A hierarchical modeling approach could prove effective—with shared foundational pathways at the population level and patient-specific modules for driver mutations. Cloud-based deployment of containerized models (e.g., using Docker/Kubernetes) could address computational bottlenecks. Furthermore, given the computational complexity of simulating individual patient models, what strategies will be employed to ensure that AIVCs can be expanded in real-world clinical settings, where time and resource constraints are critical considerations?

Finally, while the article touches on the importance of interdisciplinary collaboration, it does not fully address the practical challenges of integrating the expertise of clinicians, experimental scientists, and AI researchers [5]. The establishment of “Translational AI Units” embedding computationalists within clinical teams, supported by standardized ontologies (e.g., Open Biological and Biomedical Ontologies Foundry) and interoperable platforms (e.g., Health Level Seven International/Fast Healthcare Interoperability Resources for clinical data), could bridge this gap. How will the development of AIVCs be coordinated across these diverse fields to ensure that the models are both biologically relevant and computationally feasible? What frameworks will be established to facilitate the sharing of data, tools, and insights, particularly in light of the ethical and privacy concerns associated with patient data?

In conclusion, while Yang et al. [1] provide a visionary perspective on the potential of AIVCs in cancer research, several unresolved questions highlight the need for further exploration and collaboration. The proposed solutions—spanning stochastic modeling frameworks, hybrid multi-omics architectures, constrained generative AI, modular personalized models, and embedded translational teams—provide concrete pathways forward. Addressing these questions will be essential to realizing the full potential of AIVCs and advancing the field of oncology towards more precise and effective treatments. We look forward to seeing how these challenges are tackled in future research and how AIVCs will evolve to meet the complex demands of cancer biology.

## Data Availability

Not applicable.

## Abbreviations

AI: Artificial intelligence
AIVCs: Artificial intelligence virtual cells
